# Tumor Microenvironment Characterization in Breast Cancer Identifies Prognostic and Neoadjuvant Chemotherapy Relevant Signatures

**DOI:** 10.3389/fmolb.2021.759495

**Published:** 2021-10-11

**Authors:** Fei Ji, Jiao-Mei Yuan, Hong-Fei Gao, Ai-Qi Xu, Zheng Yang, Ci-Qiu Yang, Liu-Lu Zhang, Mei Yang, Jie-Qing Li, Teng Zhu, Min-Yi Cheng, Si-Yan Wu, Kun Wang

**Affiliations:** ^1^ Department of Breast Cancer, Cancer Center, Guangdong Provincial People’s Hospital, Guangdong Academy of Medical Sciences, Guangzhou, China; ^2^ School of Medicine, South China University of Technology, Guangzhou University Town, Guangzhou, China; ^3^ Department of Pathology, Guangdong Provincial People’s Hospital, Guangdong Academy of Medical Sciences, Guangzhou, China; ^4^ Department of Operation Room, Guangdong Provincial People’s Hospital, Guangdong Academy of Medical Sciences, Guangzhou, China

**Keywords:** breast cancer, stromal immunotype, prognosis, biomarker, neoadjuvant chemotherapy

## Abstract

Immune response which involves distinct immune cells is associated with prognosis of breast cancer. Nonetheless, less study have determined the associations of different types of immune cells with patient survival and treatment response. In this study, A total of 1,502 estrogen receptor(ER)-negative breast cancers from public databases were used to infer the proportions of 22 subsets of immune cells. Another 320 ER-negative breast cancer patients from Guangdong Provincial People’s Hospital were also included and divided into the testing and validation cohorts. CD8^+^ T cells, CD4^+^ T cells, B cells, and M1 macrophages were associated with favourable outcome (all *p* <0.01), whereas Treg cells were strongly associated with poor outcome (*p* = 0.005). Using the LASSO model, we classified patients into the stromal immunotype A and B subgroups according to immunoscores. The 10 years OS and DFS rates were significantly higher in the immunotype A subgroup than immunotype B subgroup. Stromal immunotype was identified as an independent prognostic indicator in multivariate analysis in all cohorts and was also related to pathological complete response(pCR) after neoadjuvant chemotherapy. The nomogram that integrated the immunotype and clinicopathologic features showed good predictive accuracy for pCR and discriminatory power. The stromal immunotype A subgroup had higher expression levels of immune checkpoint molecules (PD-L1, PD-1, and CTLA-4) and cytokines (IL-2, INF-γ, and TGF-β). In addition, patients with immunotype A and B diseases had distinct mutation signatures. Therefore, The stromal immunotypes could predict survival and responses of ER-negative breast cancer patients to neoadjuvant chemotherapy.

## Introduction

Breast cancer is the most common cancer in women and a leading cancer worldwide. Genomic changes in cancer cells may be used to predict prognosis and treatment responses as well as to develop new targeted therapy (Cancer Genome Altas Network, 2012; Curtis et al., 2012; Ali et al., 2014; Ji et al., 2019). Recently, it has been reported that the tumor microenvironment also played an important role in tumor progression and chemotherapy efficacy. breast cancer is composed of a mixtures of cancer cells and non-cancer cells such as stromal cells, vascular cells, and tumor-infiltrating lymphocytes (TILs), with the roles of non-cancer cells remain unclear. Among the non-cancer cells, TIL values have been reported to be associated with pathologically complete response (pCR) and prolonged overall survival (OS) in patients with breast cancer (Ladoire et al., 2008; Kitano et al., 2017; Luen et al., 2017), it also could be used as a drug target, but the roles of specific immune cells have not been well clarified. To better understand the diverse immune cells of breast cancer to the response of treatment and construct the classification of stromal immunotype for survival prediction, we enumerate immune cells in a way that accounts for the breadth of their specialized functions, and to reliably investigate the interaction of the immune response with breast cancer, and finally find the effective parameters to support the personalized therapy.

Therefore, the aim of this study was to quantify the composition of immune cells and investigate their relationships with responses to neoadjuvant chemotherapy and survival of breast cancer patients.

## Patients and Methods

### Study Population

The present study was approved by the Ethics Committee of the Guangdong Provincial People’s Hospital. Three independent cohorts of patients with breast cancer were included. The training cohort was comprised of 1,502 estrogen receptor(ER)-negative breast cancer patients with gene expression data from the public studies, details of patients from public studies can be found in corresponding publications(Supplementary Table S1), some data were compiled and created as previously described (Haibe-Kains et al., 2012; Ali et al., 2016). some data were downloaded from Gene Expression Omnibus and ArrayExpress(TCGA and METABRIC). After excluding patients with comorbidities (e.g., other malignant tumors), incomplete follow-up data, and metastatic disease, 320 patients with pathologically diagnosed ER-negative breast cancer using needle core biopsy at the Guangdong Provincial People’s Hospital between June 2009 and December 2015 were selected and randomly divided into the testing cohort (*n* = 218) and the validation cohort (*n* = 102) by using computer-generated random numbers. pCR was defined as the absence of any residual invasive carcinoma or DCIS on pathologic review of a surgical specimen following neoadjuvant chemotherapy. The study design is shown in Supplementary Figure S1. Informed consent was signed by each patient to allow the use of their data in clinical researches.

### Immunoscore Establishment

**FIGURE 1:**
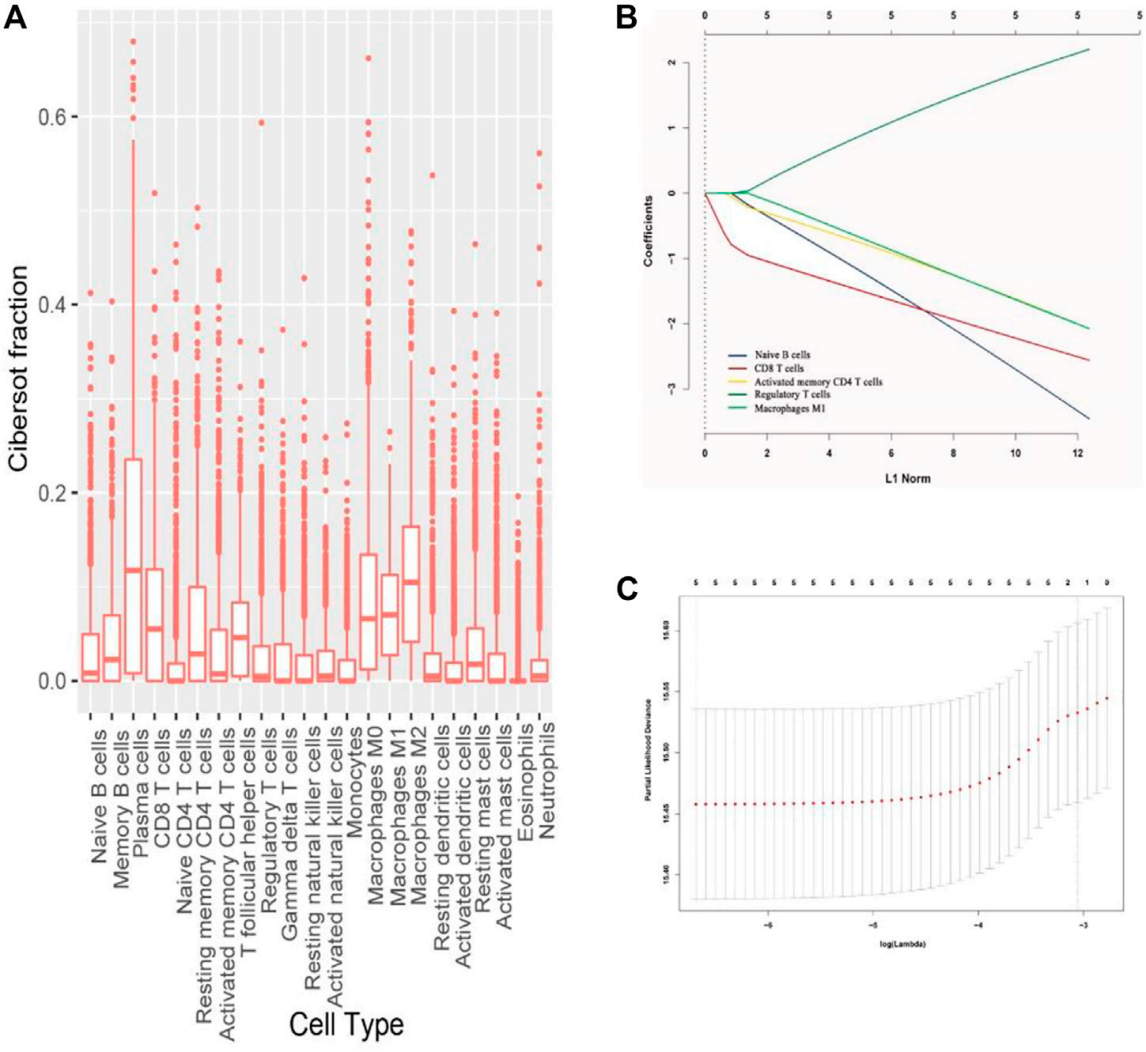
**(A)** Fraction data of the 22 types of immune cells. **(B)** LASSO coefficient profiles of the five selected stromal features for overall survival. **(C)** Partial likelihood deviance of the LASSO coefficient profiles for overall survival.

Normalized gene expression data were used to infer the relative proportions of 22 types of infiltrating immune cells using the CIBERSORT algorithm. For the TCGA dataset, RNA sequencing data were transformed using voom, converting count data to values more similar to those resulting from microarrays (Cancer Genome Altas Network, 2012; Law et al., 2014). The CIBERSORT is a well-designed method for the analysis of microarray gene expression profiles (Ali et al., 2016; Li et al., 2019). The LM22 file covers 547 genes that can be used to accurately discriminate 22 distinct functional subsets of immune cells and activation states including seven T cell types, naive and memory B cells, plasma cells, NK cells, and myeloid subsets. So in our study, we used the CIBERSORT and the LM22 gene signature file to estimate the fractions of diverse immune cells in the patients. The associations of immune cells with the OS and disease-free survival (DFS) of patients in the training cohort were analyzed using the estimated fractions by univariate analysis. The COX regression analysis with the least absolute shrinkage and selection operator (LASSO) model was used to develop an immunoscore for the classification of immunotypes A and B breast cancer (Figures 1A–C).

### Immunohistochemistry and Evaluation of Immunostaining

The identified survival-associated immune cells were stained with different antibodies in the testing and validation cohorts. IHC was performed as described in our previous studies (Ji et al., 2015; Ji et al., 2018). The following markers were used: CD4 (for CD4^+^ T cells, ab133616, Abcam, MA, United States), CD8 (for CD8^+^ T cells, ab93278, Abcam, MA, United States), CD20 (for B cells, ab78237, Abcam, MA, United States), CD80 (for M1 macrophages, ab134120, Abcam, MA, United States), and FOXP3 (for regulatory T cells, ab215206, Abcam, MA, United States). The following antibodies were used for staining: interleukin-2(IL-2,ab92381,Abcam, MA, United States), interferon-gamma (INF-γ, ab231036,Abcam, MA, United States), transforming growth factor-β2(TGF-β2, ab53778, Abcam, MA, United States), programmed cell death 1 ligand 1, (PD-L1,ab228415, Abcam, MA, United States), programmed cell death-1(PD-1, ab234444, Abcam, MA, United States), cytotoxic T lymphocyte-associated antigen-4(CTLA-4,ab228229,Abcam, MA, United States). The sections were incubated with pre-diluted primary polyclonal antibodies at 4°C overnight. The sections stained without primary antibodies were used as negative controls. The stained immune cells in three random areas of stroma and tumor core at ×200 magnification were counted. Two pathologists blinded to the clinicopathological data scored all samples independently, and the mean count was adopted.

### Statistical Analysis

The coefficients and partial likelihood deviance for each prognostic feature were calculated with the “glmnet” package in the R program. The LASSO Cox regression model was used to estimate the ideal coefficient and likelihood deviance. Restricted cubic spline regression was used to characterize the relationship between immune score and patient survival in all three cohorts. Besides, The nomogram integrating immune type and clinical parameters was constructed according to results of the multivariate logistic regression models. Based on the identified prognostic factors, the nomogram could be utilized to predict pCR after neoadjuvant chemotherapy. The discriminative capabilities of the nomogram was assessed by the area under the receiver operating characteristic curve (ROC). The differentially enriched pathways between stromal immunotypes A and B breast cancer were identified using the Gene Set Enrichment Analysis. Statistical analyses were performed using the MedCalc software (version 18; MedCalc, Mariakerke, Belgium), Stata (vension 14; StataCorp, College Station, TX), and R software packages (version 3.4.2; The R foundation for Statistical Computing, http://www.-rproject.org/).

## Results

### Stromal Immunoscore and Stromal Immunotype

**FIGURE 2:**
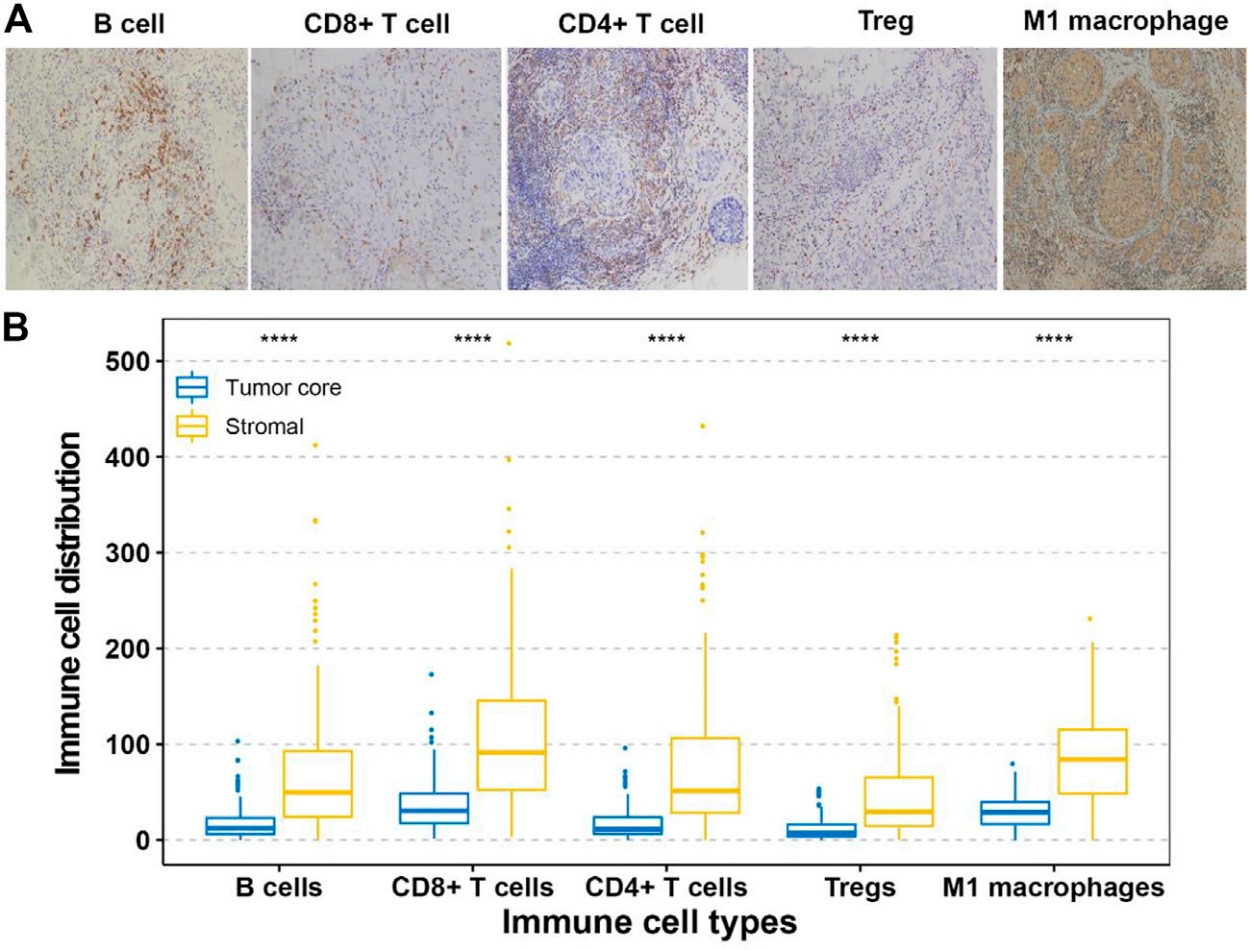
**(A)** Tumor infiltration of B cells, CD8^+^ T cells, CD4^+^ T cells, Treg cells and M1 macrophage cells in breast cancer patients. **(B)** Tumor infiltration density of B cells, CD8^+^ T cells, CD4^+^ T cells, Treg cells and M1 macrophage cells in testing and validation cohorts.

The fractions of the 22 types of immune cells are showed in Figure 1. Among them, five types (CD8^+^ T cells, CD4^+^ T cells, B cells, M1 macrophages, and Treg cells) were significantly associated with OS and DFS (Supplementary Figure S2), so we chose these five types as research cells. In the testing and validation cohorts, the mean densities of B cells, CD8^+^ T cells, CD4^+^ T cells, Treg cells, and M1 macrophages in the stroma were higher than the densities in tumor core (38.320, 77.234, 45.291, 26.291, and 71.248 cells/mm^2^ Vs 9.580, 25.745, 10.065,6.5728, and 24.568 cells/mm^2^, respectively). (Figure 2). The immune cell infiltration in the stroma was significantly associated with the OS of ER-negative breast cancer (Supplementary Table S2). The representative images of these immune cells are shown in Figure 2. We built prognostic classifiers by using the LASSO COX model in the training cohort (Supplementary Table S1; Figure 1) and calculated the coefficients using the following formulas: IS = 2.202 × Treg cell count—3.455 × B cell count—2.559 × CD8^+^ T cell count—2.077 × CD4^+^ T cell count—2.074 × M1 macrophage count. Therefore, We classified patients into immunotype A and immunotype B groups based on the median immunoscore. Clinical characteristics of patients with breast cancer of stromal immunotypes A and B in the training cohort and the testing and validation cohorts did not vary significantly (Table 1).

**TABLE 1:** Clinical characteristics of patients according to the stromal immunotype in the training, testing and validation cohorts.

Variables	Training cohort (*N* = 1,502)				Testing cohort (*N* = 218)				Validation cohort (*N* = 102)			
	*N*	Immunotype A (%)	Immunotype B (%)	*p*	*N*	Immunotype A (%)	Immunotype B (%)	*p*	*N*	Immunotype A (%)	Immunotype B (%)	*p*
**Age(yrs)**		52.56 ± 12.20	53.02 ± 12.63	0.495		53.11 ± 11.1	53.21 ± 12.6	0.954		51.86 ± 12.71	52.86 ± 11.30	0.671
**Axillary lymph nodes**				0.293				0.643				0.251
Positive	398	190 (12.6%)	208 (13.8%)		92	46 (21.1%)	46 (21.1%)		47	22 (21.6%)	25 (24.5%)	
Negative	1,104	561 (37.4%)	543 (36.2%)		126	67 (30.7%)	59 (27.1%)		55	32 (31.4%)	23 (22.5%)	
**TNM stage**				0.101				0.477				0.561
I-II	1,108	540 (36.0%)	568 (37.8%)		183	87 (39.9%)	76 (34.9%)		88	41 (40.2%)	34 (33.3%)	
III-IV	394	211 (14.0%)	183 (12.2%)		35	26 (11.9%)	29 (13.3%)		14	13 (12.7%)	14 (13.8%)	
**Tumor size**				0.241				0.2860				0.300
0–20 mm	462	246 (16.4%)	216(14.4%)		67	18 (8.3%)	18 (8.3%)		29	7 (6.9%)	11 (10.8%)	
21–50 mm	782	381 (25.4%)	401 (26.7%)		122	78 (35.8%)	63 (28.9%)		48	39 (38.2%)	28 (27.5%)	
>50 mm	258	124(8.3%)	134(8.8%)		29	17 (7.8%)	24(10.9%)		25	8 (7.8%)	9 (8.8%)	
**HER2 status by Fish**				0.101				0.056				0.168
No amplification	1,057	543 (36.2%)	514 (34.2%)		161	97 (44.5%)	64 (29.4%)		84	23 (22.5%)	27 (26.5%)	
Ampification	445	208 (13.8%)	237 (15.8%)		57	26 (11.9%)	31 (14.2%)		18	31 (30.4%)	21 (20.6%)	

### Association of Stromal Immunotype With Patient Survival

**FIGURE 3:**
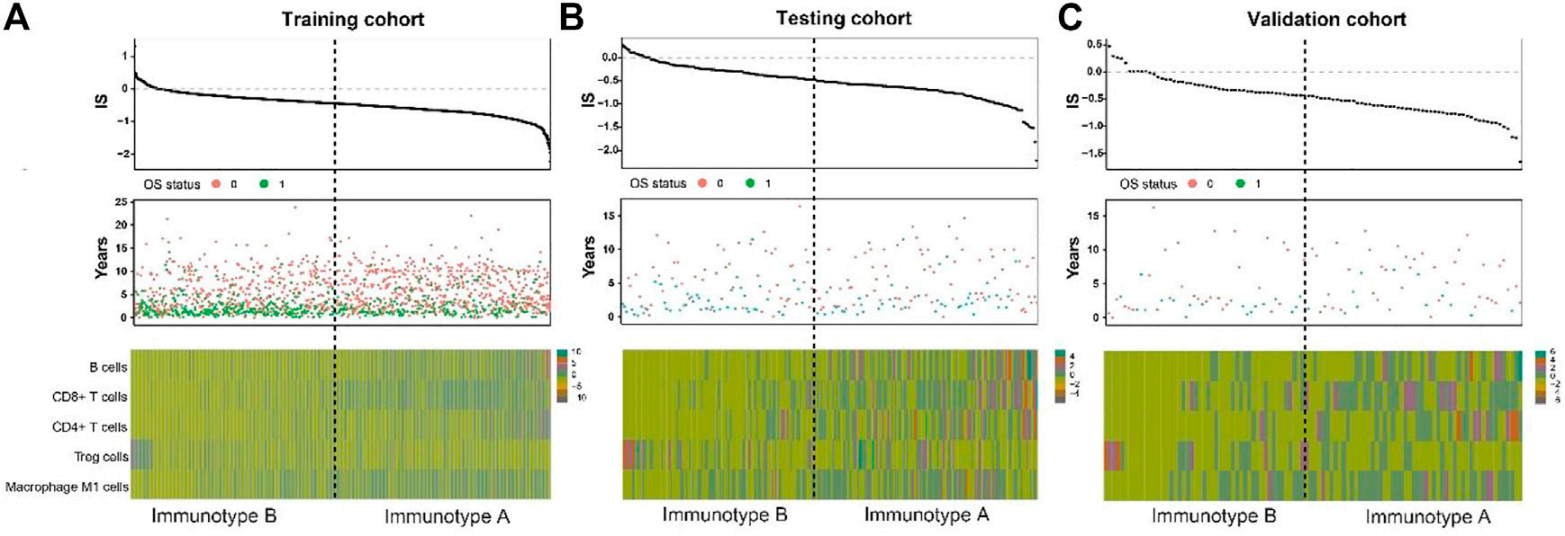
Correlate immunotype with overall survival in training cohort, testing cohort and validation cohort. Hierarchical tree structure classifying the patients of the training cohort **(A)** the patients of the testing cohort. **(B)** and the patients of the validation cohort. **(C)** according to the levels of five immune cell cluster features: high expression (green) and low expression (brown). OS status showed 0: survival; 1: death.

**FIGURE 4:**
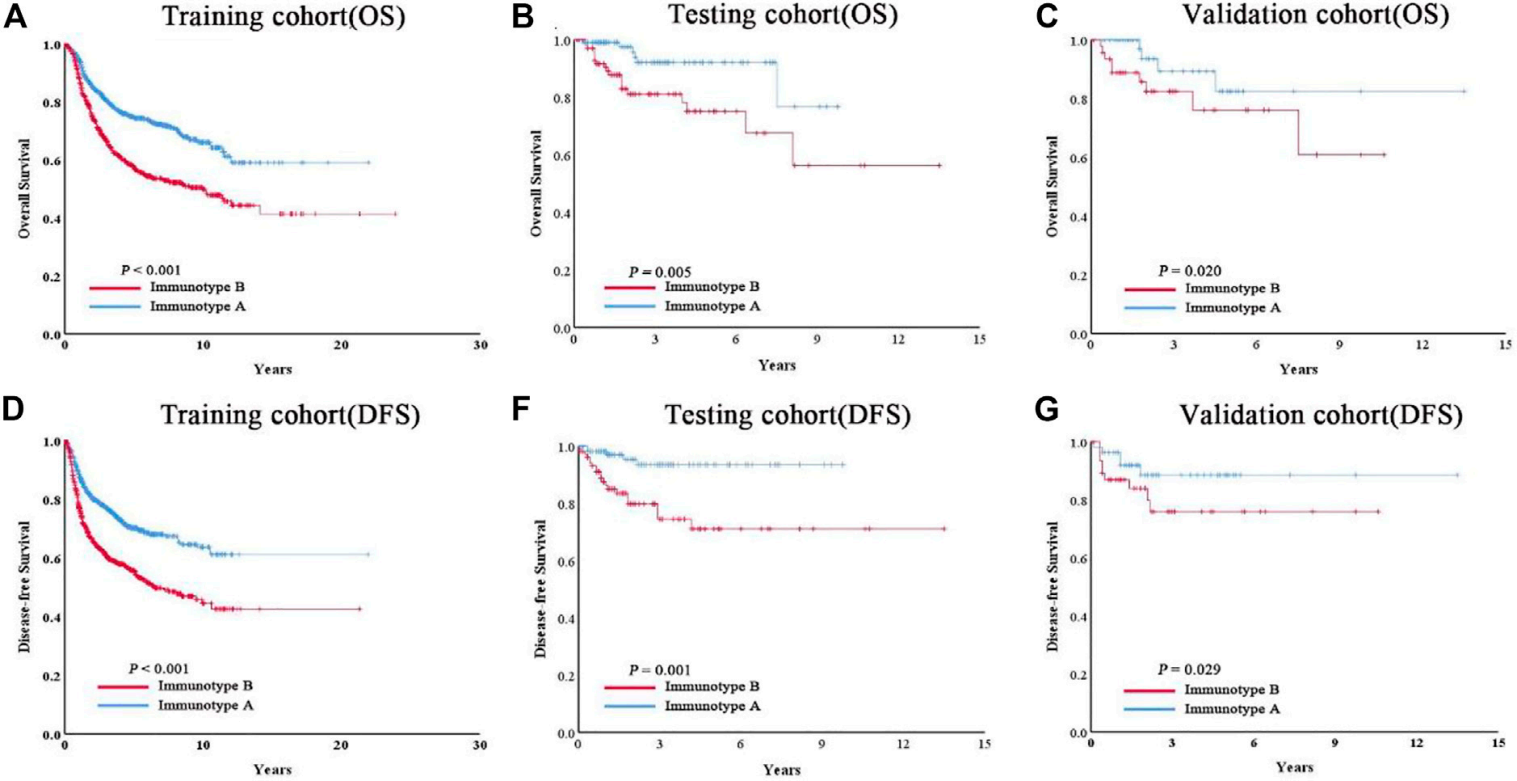
Patients with immunotype B had significantly worse overall survival **(A–C)** and disease-free survival **(D–F)** than patients with immunotype A in all three cohorts.

As shown in Figure 3, tumors with high immunoscore generally contained increased Treg cells and reduced B cells, CD8^+^ T cells, CD4^+^ T cells, and M1 macrophages. Therefore, patients in each of the three cohorts were divided into a stromal immunotype A subgroup (CD8^+^ T cells^high^, CD4^+^ T cells^high^, B cells^high^, M1 macrophages^high^, and Treg cells^low^) and a stromal immunotype B subgroup (CD8^+^ T cells^low^, CD4^+^ T cells^low^, B cells^low^, M1 macrophages^low^, and Treg cells^high^). Patients in the immunotype B subgroup had a higher death rate as well as shorter DFS and OS than patients in the immunotype A subgroup. The 10-years DFS and OS rates were significantly higher in patients immunotype A disease than in those with immunotype B disease in the training (DFS: 63.7 vs. 44.5%, *p* < 0.001; OS: 66.0 vs. 49.9%, *p* < 0.001), testing (DFS: 40.2 vs. 17.6%, *p* = 0.025; OS: 32.0 vs. 20.7%, *p* = 0.034), and validation cohorts (DFS: 66.5 vs. 27.0%, *p* = 0.020; OS: 61.7 vs. 34.4%, *p* = 0.029) (Figure 4).

### Association of Stromal Immunotype With Response to Neoadjuvant Chemotherapy

**FIGURE 5:**
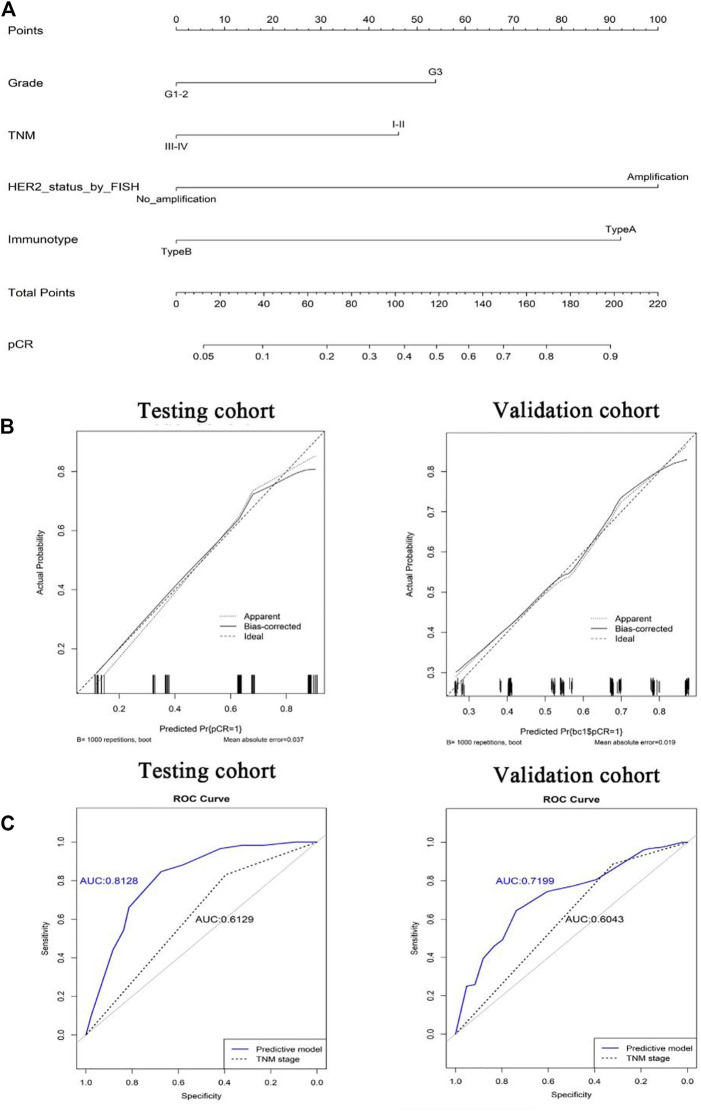
Nomograms (for pCR) that integrated the immunotype and clinicopathologic risk factors **(A)**. Calibration curves showing the discriminatory power of the nomogram for pCR in testing and validation cohorts **(B)**. ROC curves showing the predictive accuracy of the nomogram for pCR and the predictive accuracy was higher than that of the TNM staging system in both testing and validation cohorts **(C)**.

To quantitatively predict responses to neoadjuvant chemotherapy, we constructed a nomogram which integrated both stromal immunotype and clinicopathological factors using the data from the testing cohort and validated it using the data from the validation cohort. First, we analyzed the relationships between pCR and clinicopathologic characteristics and found that the rate of pCR was associated with tumor size, histologic grade, HER2 status, TNM stage, immunotype in the testing cohort and the validation cohort (all *p* < 0.05) (Table 2). Multivariate logistic analyses in both cohorts showed that histologic grade, TNM stage, HER2 status, and immunotype were independent predictors for pCR (all *p* < 0.05) (Table 3). Therefore, we constructed a nomogram using these factors (Figure 5). The calibration plots and ROC curve (AUC: 0.8128) showed that the derived nomogram performed well, with a high pCR-predictive ability in the validation cohort (AUC: 0.7199). Interestingly, the predictive accuracy of our nomogram was higher than that of the TNM staging system in both cohorts (AUC: 0.6129 and 0.6043).

**TABLE 2:** The relationship between pCR and clinicopathologic characters in testing cohort and validation cohort.

Variables	Testing cohort				Validation cohort			
	Cases	pCR	nonpCR	*p* value	Cases	pCR	nonpCR	*p* value
**Age(yrs)**				0.393				0.119
**>60**	36	19 (8.7%)	17(7.8%)		21	9(8.8%)	12(11.8%)	
**≤60**	182	110(50.5%)	72(33.0%)		81	50(49.0%)	31(30.4%)	
**Tumor size**				<0.001				0.039
**T1-T2**	177	116(53.2%)	61(30.0%)		84	53(52.0%)	32(31.4%)	
**T3-T4**	41	13(6.0%)	28(10.8%)		18	6(5.9%)	11(10.7%)	
**Nodal status**				0.040				0.487
**negative**	89	60(27.5%)	29(13.3%)		42	26(25.5%)	16(15.7%)	
**positive**	129	69(31.7%)	60(27.5%)		60	33(32.4%)	27(26.4%)	
**Histologic grade**				0.019				0.013
**G1-G2**	92	46(21.1%)	46(21.1%)		47	21(20.6%)	26(25.5%)	
**G3**	126	83(38.1%)	43(19.7%)		55	38(37.3%)	17(16.6%)	
**HER2 status by FISH**				0.008				0.005
**No amplification**	99	49(22.5%)	50(22.9%)		50	22(21.6%)	28(27.5%)	
**Amplification**	119	80(36.7%)	39(17.9%)		52	37(36.3%)	15(14.6%)	
**TNM**				0.001				0.011
**I-II**	163	107(49.1%)	56(25.7%)		75	49(48.0%)	26(25.5%)	
**III-IV**	55	22(10.1%)	33(15.1%)		27	10(9.8%)	17(16.7%)	
**Risk score**				<0.001				
**Immunotype A**	113	82(37.6%)	31(14.2%)		54	39(38.2%)	15(14.7%)	0.002
**Immunotype B**	105	47(21.6%)	58(26.6%)		48	20(19.6%)	28(27.5%)	

**TABLE 3:** Multivariate logistic regression analysis.

Variables	Testing cohort				Validation cohort			
	βvalue	OR value	95%CI	*p* value	βvalue	OR value	95%CI	*p* value
**Grade**	0.843	2.323	1.196–4.512	0.013	1.720	5.583	1.808–17.240	0.003
**TNM**	−1.329	0.265	0.121–0.580	0.001	−1.657	0.191	0.059–0.614	0.005
**HER2 status By Fish**	−1.105	0.331	0.169–0.647	0.001	−2.137	0.118	0.036–0.387	<0.001
**Immunotype**	−0.904	0.405	0.218–0.750	0.004	−1.657	0.191	0.059–0.614	0.005

### Identification of Stromal Immunotype-Associated Biological Pathways and Immune Checkpoint Molecules

**FIGURE 6:**
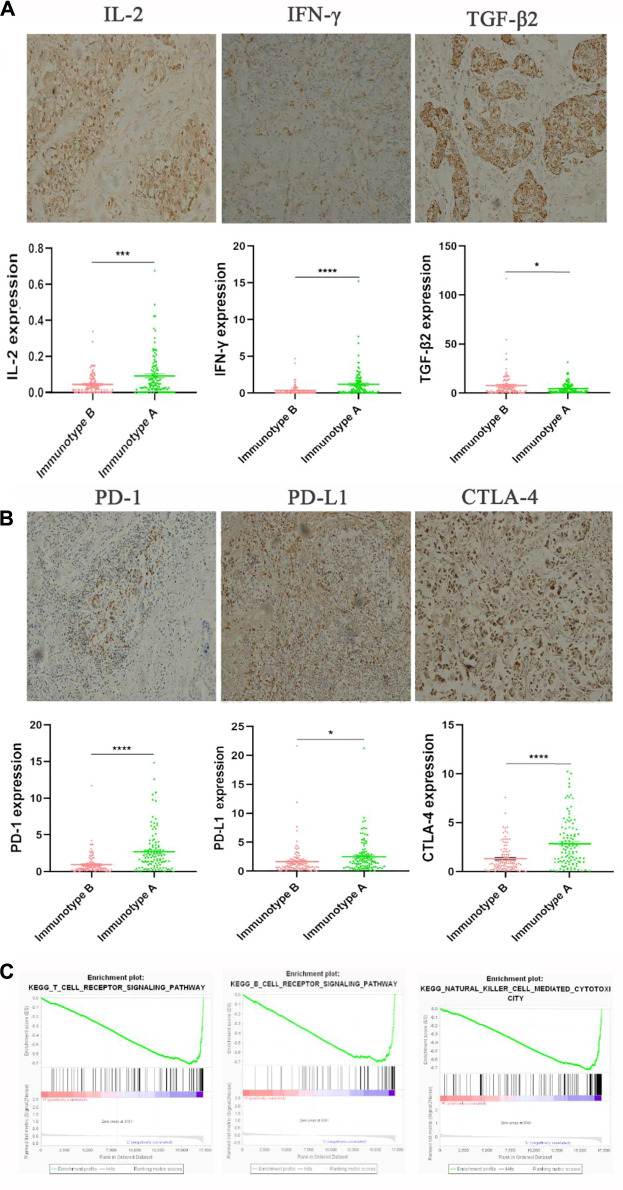
The expression of immunomarkers and signal pathway of stromal immunotypes. The representative immunohistochemistry images of IL-2, IFN-γ, TGF-β2 **(A)**. The associations of immunotype with the immune check-point markers **(B)**. Three enriched biology pathways related with different stromal immunotypes **(C)**.

By IHC staining, we found three important cytokines (IL-2, INF-γ, and TGF-β) that were differently expressed between immunotype A and B subgroups. IL-2, INF-γ expression was significantly higher in the immunotype A subgroup, and TGF-β expression was significantly higher in the immunotype B subgroup (Figure 6). Furthermore, the expression levels of several immune checkpoint molecules (PD-L1, PD-1, and CTLA-4) were significantly higher in the immunotype A subgroup than in the immunotype B subgroup (Figure 6). The Gene Set Enrichment Analysis showed that the T-cell receptor signaling pathway, B-cell receptor signaling pathway, and NK cell-mediated immunity were highly enriched in the immunotype A subgroup (Figure 6).

## Discussion

Tumor microenvironment is involved in not only the progression but also the responses to anticancer therapies and outcomes of breast cancer (Yu et al., 2017). TILs in the microenvironment can be used to monitor the immune response and are important in predicting treatment responses in many cancers (Asano et al., 2016). Denkert et al. (Denkert et al., 2018) have demonstrated that increased TIL concentration was associated with good response to neoadjuvant chemotherapy in all molecular subtypes of breast cancer and with a survival benefit in HER2-positive and triple-negative breast cancer. TILs include various types of immune cells. Some types of TILs have been shown to be associated with survival or proliferation of cancer cells, for example, high Treg cell count was related to poor clinical outcomes (McCoy et al., 2015). However, not all TILs have definitive prognostic values. Therefore, we applied the CIBERSORT algorithm (Ali et al., 2016) to estimate the relative proportions of 22 distinct functional subsets of immune cells in ER-negative breast cancer patients. CD8^+^ T cells, CD4^+^ T cells, B cells, M1 macrophages, and Treg cells with significant prognostic values were selected using univariate cox regression. Then five selected types of immune cells were used to build a stromal immunotype. The immunotype not only indicated the bio-immunological information of breast cancer but also showed the location information of immune cell infiltration, giving us a clear preview of immune contexture. With further analysis, tumors with high immunoscore (immunotype B) consisted of high levels of Treg cells and low levels of B cells, CD8^+^ T cells, CD4^+^ T cells, and M1 macrophages, while tumors with low immunoscore (immunotype A) consisted of low levels of Treg cells and high levels of B cells, CD8^+^ T cells, CD4^+^ T cells, and M1 macrophages. Patients in the immunotype B subgroup had significantly shorter OS and DFS than those in the immunotype A subgroup.

Interleukin-2 (IL-2), a cell growth factor in the immune system, can promote the proliferation of Th0 and CTL. IFN-γ, a multifunctional cytokine, plays an important role in promoting antigen presentation and enhancing the activity of macrophages and natural killer cells. TGF-β regulates tumor-stroma interactions, angiogenesis, and metastasis and shows an immunosuppressive function, inhibiting the proliferation and activity of CTL and B cells, while promoting the proliferation of M2 macrophages (Sia et al., 2017; Xue et al., 2020). Interestingly, in the present study, we found that IL-2 and IFN-γ were increased in the immunotype A patients, whereas the TGF-β-mediated immunosuppression pathway was elevated in immunotype B patients. What’s more, the Gene Set Enrichment Analysis showed that the B-cell receptor signaling pathway, T cell-mediated pathway, and NK cell-mediated immunity were highly enriched in immunotype A patients. All these findings suggest that immunotype A indicates a hyperimmune state, with a strong tumor-immune cell interaction. CD4^+^ T-cells in immunotype A may play an important role in recruiting and modulating cytotoxic T-cells in antitumor immunity and CD4^+^ T-cell activity contributes to the full function of CD8^+^ cytotoxic T-cells, which is effective for tumor control. While immunotype B indicates a status of immunosuppression for the level of Tregs. These may also explain the prognostic heterogeneity in patients with different immunotypes. In addition, we found that immunotype A was associated with high expression levels of immune checkpoint molecules and cytokines, suggesting that immune checkpoint inhibitor might work well in this subgroup. Therefore, the stromal immunotype might be used to predict responses to immunotherapy.

Recently, many studies focused on biomarkers that were highly associated with treatment responses such as chemotherapy (Lee et al., 2019; Al Amri et al., 2020; Jiao et al., 2020; Valdés-Ferrada et al., 2020; Zhao et al., 2020), and most of them focused on signatures of genes, microRNAs, lncRNAs, and epigenetic biomarkers for the prediction of long-term survival in patients with tumor. However, these signatures cannot be widely used in clinic because of the variability in gene sequencing methods, the inconvenience in the use of assay platforms, and the requirement for specialized analyses. In the present study, we quantified immune cells using IHC staining, which might be easily applied in clinical practice. Moreover, breast cancer has heterogeneous prognosis. To better predict the responses after neoadjuvant chemotherapy, we constructed a nomogram combining clinical characteristics and immunotype. The calibration plots and ROC curve showed that the derived nomogram outperformed the TNM staging system (the 7^th^ edition).

The present study had several limitations. First, as a retrospective study with relatively small sample sizes, our results need to be validated in a prospective study with larger cohorts. Second, not all of the 22 subsets of immune cells were analyzed. We selected only five subsets using the CIBERSORT method. Other important immunotypes such as neutrophil infiltration might also be valuable, but were not analyzed in the present study. The immunotype classification needs to be optimized with more important immune markers being identified. Third, the underlying mechanisms of the relationship between immunotype and patient prognosis were not well investigated. The role of the immune profile in the development and invasion of breast cancer needs to be further investigated.

In conclusion, immune cell infiltration in breast cancer is associated with prognosis. We defined two immunotypes by integrating the indicators of the immune cell infiltration, which could be used to predict survival and responses of breast cancer patients to neoadjuvant treatment. The immunotypes might also have significant implications in immunotherapy for the patients who are insensitive to chemotherapy.

## Data Availability

The original contributions presented in the study are included in the article/Supplementary Material, further inquiries can be directed to the corresponding author.
