# Supplementary material for: Tumor Microenvironment Characterization in Breast Cancer Identifies Prognostic and Neoadjuvant Chemotherapy Relevant Signatures
Source: Front Mol Biosci. 2021 Oct 11;8:759495. doi: 10.3389/fmolb.2021.759495 (PMC8544945; doi:10.3389/fmolb.2021.759495)
Supplement: Supplementary file 1 [file DataSheet1.docx]

Table S1 The public database in the studies involved in our paper.

| Study name | Accession number/Source | Platform | PMID |
| --- | --- | --- | --- |
| DFHCC | GEO: GSE19615 | Affymetrix HGU | 20098429 |
| DFHCC2 | GEO: GSE18864 | Affymetrix HGU | 20100965 |
| DFHCC3 | GEO: GSE3744 | Affymetrix HGU | 16473279 |
| EMC2 | GEO: GSE12276 | Affymetrix HGU | 19421193 |
| IPC | GEO: GSE17907 | Affymetrix HGU | 20932292 |
| IRB/JRH/NUH | GEO: GSE45255 | Affymetrix HGU | 23618380 |
| KOO-1 | GEO: GSE20685 | Affymetrix HGU | 21501481 |
| MAINZ | GEO: GSE11121 | Affymetrix HGU | 18593943 |
| MDACC Hatzis | GEO: GSE25066 | Affymetrix HGU | 21558518 |
| MGH | GEO: GSE1378 | Arcturus 22k human oligonucleotide microarray | 15193263 |
| MSK | GEO: GSE2603 | Affymetrix HGU | 16049480 |
| Montpellier | GEO: GSE9893 | MLRG Human 21K V12.0 | 18347175 |
| NCI | Authors’ website | In-house cDNA array | 12917485 |
| NKI | Rosetta Inpharmatics | Agilent | 12490681 |
| NTUH | GEO: GSE33926 | Agilent-012097 Human 1A Microarray | 23049873 |
| PNC | GEO: GSE20711 | Affymetric HGU | 21910250 |
| STK | GEO: GSE1456 | Affymetrix HGU | 16280042 |
| STNO2 | Stanford Microarray DB | In-house cDNA array | 12829800 |
| TAM | GEO: GSE6532/GSE9195 | Affymetrix HGU | 17401012 |
| TCGA | TCGA data portal | RNA-seq | 26451490 |
| TOP trial | GEO: GSE16446 | Affymetrix HGU | 21422418 |
| TRANSBIG | GEO: GSE7390 | Affymetrix HGU | 17545524 |
| UHN | GEO: GSE45725 | Illumina HumanRef-8 v3.0 expression beadchip | 24996446 |
| UNC4 | UNC DB | Agilent | 20813035 |
| UNT | GEO: GSE2990 | Affymetrix HGU | 16478745 |
| UPP | GEO: GSE3494 | Affymetrix HGU | 16141321 |
| Uppsala | GEO: GSE4922 | Affymetrix HGU | 17079448 |
| VDX | GEO: GSE2034/GSE5327 | Affymetrix HGU | 17420468 |
| VDX3 | GEO: GSE12093 | Affymetrix HGU | 18821012 |
| WIMM | GEO: GSE22219 | Illumina humanRef-8 v1.0 expression beadchip | 21737487 |


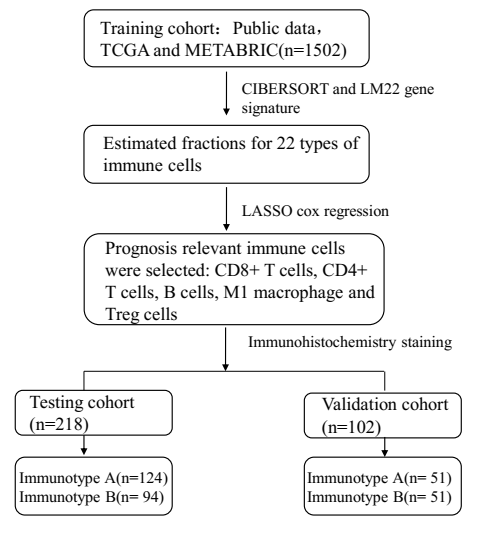


Fig. S1 The study design of this study. Patients in the public database, TCGA and METABRIC cohort were used as the training cohort. By CIBERSORT method and LM22 gene signatures, and finally five immune cells were selected as the prognosis relevant features. To validate the results observed in the training cohort, immunohistochemistry staining of these five immune cells was performed for patients in the testing and validation cohorts.


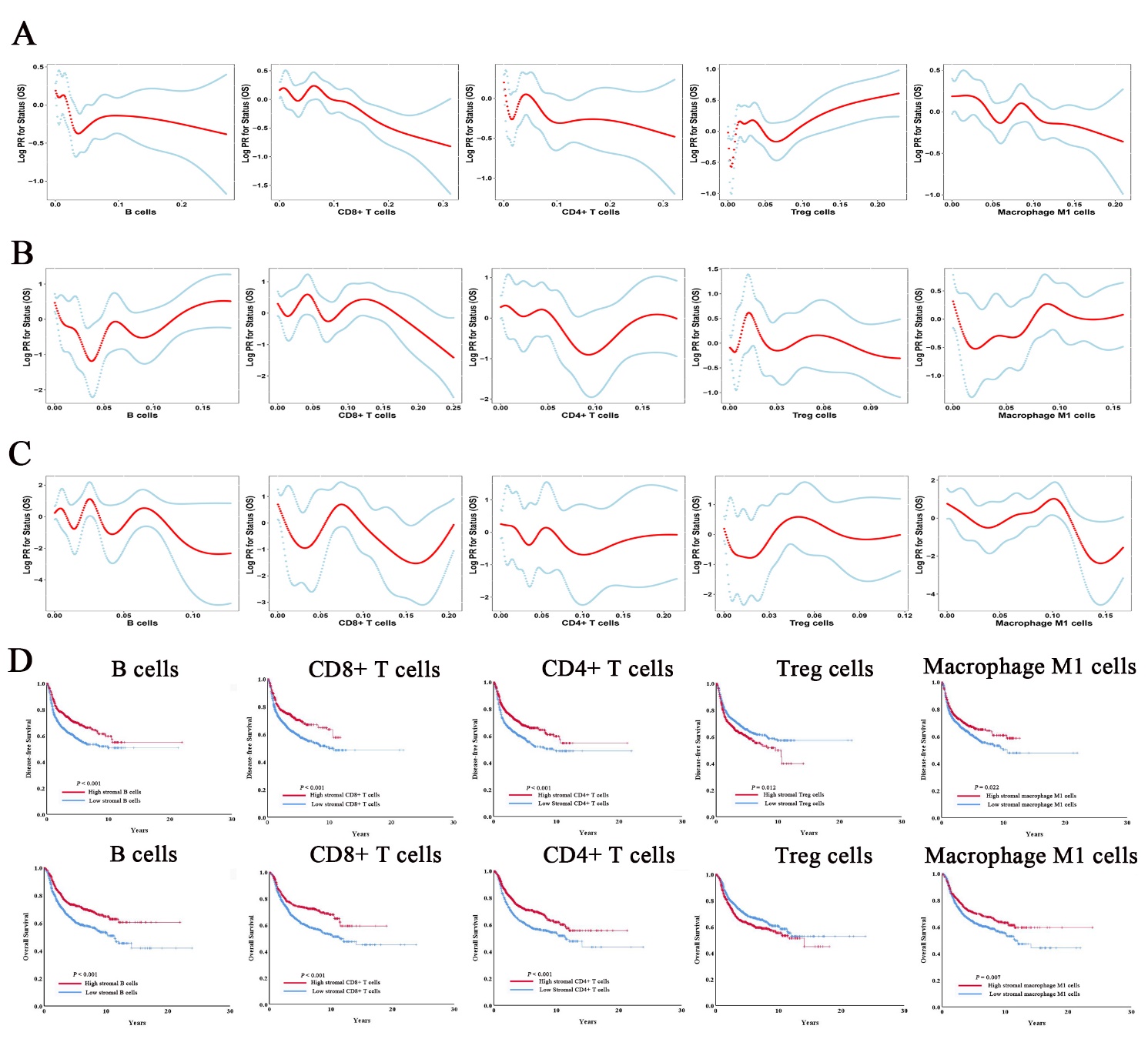


Figure S2 The restricted cubic spline(A-C) and DFS/OS(D) of the selected immune cells(B cells, CD8+ T cells, CD4+ T cells, Treg cells, and M1 macrophages) in the training cohort.

| **Table S2. Univariate Cox Regression Analysis of 5 immune phenotypes and overall survival** | | | | |
| --- | --- | --- | --- | --- |
| **Covariate** | ***P*** | **Hazard Ratio** | **95% CI of Hazard Ratio** |  |
| **Training cohort** |  |  |  |  |
| CD8+ T cells | <0.0001 | 0.064 | 0.018 to 0.234 |  |
| CD4+ T cells | 0.002 | 0.072 | 0.013 to 0.382 |  |
| B cells | 0.003 | 0.040 | 0.005 to 0.340 |  |
| M1 macrophage cells | 0.006 | 0.090 | 0.016 to 0.502 |  |
| Treg cells | 0.005 | 10.971 | 2.059 to 58.444 |  |
| **Testing cohort**  Stromal CD8+ T cells  Stromal CD4+ T cells  Stromal B cells  Stromal M1 macrophage cells  Stromal Treg cells  **Validation cohort**  Stromal CD8+ T cells  Stromal CD4+ T cells  Stromal B cells  Stromal M1 macrophage cells  Stromal Treg cells | 0.035  0.019  0.027  0.024  0.004  0.019  0.023  0.039  0.033  0.006 | 0.055  0.002  0.024  0.091  7.413  0.015  0.008  0.000  0.003  5.894 | 0.004 to 0.818  0.000 to 0.360  0.001 to 0.653  0.011 to 0.727  1.919 to 28.633  0.000 to 0.509  0.000 to 0.510  0.000 to 0.573  0.000 to 0.623  3.963 to 8.325 |  |
